# Microbiota–Neuroinflammation Crosstalk in Primary Brain Tumors: Focus on Glioblastoma

**DOI:** 10.1002/brb3.71648

**Published:** 2026-08-05

**Authors:** Tareq Nayef AlRamadneh, Renuka Jyothi S, Priya Priyadarshini Nayak, Anima Nanda, Shaker Al Hasnaawei, Akanksha Bhatt, Ashish Singh Chauhan, Siya Singla, Mehrdad Nourizadeh

**Affiliations:** ^1^ Faculty of Allied Medical Sciences, Hourani Center For Applied Scientific Research Al‐Ahliyya Amman University Amman Jordan; ^2^ Department of Biotechnology and Genetics School of Sciences JAIN (Deemed to Be University) Bangalore Karnataka India; ^3^ Department of Medical Oncology IMS and SUM Hospital, Siksha ‘O’ Anusandhan Bhubaneswar Odisha India; ^4^ Department of Biomedical Sathyabama Institute of Science and Technology Chennai Tamil Nadu India; ^5^ College of Pharmacy The Islamic University Najaf Iraq; ^6^ Department of Medical Analysis, Medical Laboratory Technique College The Islamic University of Al Diwaniyah Al Diwaniyah Iraq; ^7^ Department of Pharmacy Graphic Era Hill University Dehradun India; ^8^ Centre For Promotion of Research Graphic Era Deemed to Be University Dehradun Uttarakhand India; ^9^ Uttaranchal Institute of Pharmaceutical Sciences, Division of Research and Innovation Uttaranchal University Dehradun Uttarakhand India; ^10^ Centre For Research Impact Outcome, Chitkara University Institute of Engineering and Technology Chitkara University Rajpura Punjab India; ^11^ Neuroscience Research Center Tabriz University of Medical Sciences Tabriz Iran

**Keywords:** glioblastoma, gut–brain axis, immunotherapy, microbiota‐derived metabolites, neuroinflammation, tumor microenvironment

## Abstract

**Purpose:**

Glioblastoma (GBM) is the most aggressive primary brain tumor in adults and remains difficult to treat because of diffuse invasion, immunosuppression, metabolic adaptability, and therapy resistance. This review evaluates how gut microbiota and microbiota‐associated neuroinflammatory signaling may contribute to GBM biology and therapeutic response.

**Method:**

We synthesized mechanistic, preclinical, translational, and emerging clinical evidence on microbiota‐neuroinflammation interactions in GBM. The review focused on gut‐brain axis pathways, microbial metabolites, blood‐brain barrier (BBB) regulation, glial and myeloid immune activity, tumor‐associated microbial signatures, microbial peptide‐HLA presentation, and microbiome‐informed biomarker or therapeutic strategies.

**Finding:**

Current evidence suggests that microbiota‐related signals may influence GBM through systemic immune modulation, short‐chain fatty acids, tryptophan‐derived metabolites, polyamines, BBB effects, and altered microglial and tumor‐associated myeloid cell function. Polyamine metabolism may sustain myeloid‐cell‐mediated immunosuppression in the acidic GBM tumor microenvironment, whereas microglial GLUT5‐dependent fructose metabolism may limit inflammatory antigen presentation and adaptive antitumor immunity. Sequencing‐based studies have reported bacterial and fungal nucleic acid signatures in brain tumor specimens, but these findings require careful interpretation because of low biomass, contamination risk, and methodological variability. Preclinical models further indicate that microbiome modulation can alter inflammatory tone, tumor growth, immune‐cell infiltration, and response to immune checkpoint blockade.

**Conclusion:**

Microbiota‐regulated neuroinflammation is a biologically plausible contributor to GBM progression, immune suppression, and treatment resistance. However, most evidence remains preclinical or early translational. Well‐controlled, spatially resolved, multi‐omic studies are required before microbiome‐based biomarkers or interventions can be clinically implemented for patient stratification and future precision clinical neuro‐oncology applications.

## Graphical abstract

Microbiota–neuroinflammation crosstalk in GBM.

The schematic summarizes how gut microbiota and microbiota‐derived signals may influence GBM biology through gut–brain signaling, microbial metabolites, systemic immune modulation, BBB‐related pathways, tumor‐associated microbial signals, and immune‐metabolic changes within the GBM microenvironment. These interactions may contribute to immune suppression, tumor progression, therapy resistance, and future microbiome‐informed translational strategies.

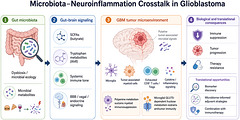

**Abbreviations**
AhR: aryl hydrocarbon receptor
BBB: blood–brain barrier
CCL2: chemokine (C‐C motif) ligand 2
CCL22: chemokine (C‐C motif) ligand 22
CNS: central nervous system
CSF: cerebrospinal fluid
CSF‐1: colony‐stimulating factor 1
FMT: fecal microbiota transplant
GABA: gamma‐aminobutyric acid
GBA: gut–brain axis
GBM: glioblastoma
GF: germ‐free (mice)
HDAC: histone deacetylase
HLA: human leukocyte antigen
ICIs: immune checkpoint inhibitors
IFN‐γ: interferon‐gamma
IL‐10: interleukin‐10
IL‐1β: interleukin‐1 beta
IL‐6: interleukin‐6
MHC: major histocompatibility complex
NF‐Κb: nuclear factor‐kappa B
PD‐L1: programmed death‐ligand 1
PRRs: pattern recognition receptors
SCFAs: short‐chain fatty acids
TAAs: tumor‐associated antigens
TAMs: tumor‐associated macrophages
TGF‐β: transforming growth factor‐beta
TILs: tumor‐infiltrating lymphocytes
TME: tumor microenvironment
TNF‐α: tumor necrosis factor‐alpha
Tregs: regulatory T cells

## 1 Introduction

Glioblastoma (GBM), currently classified as GBM, IDH‐wildtype, CNS WHO grade 4, is the most aggressive adult‐type diffuse glioma and one of the most lethal primary brain tumors in adults (Louis et al. 2021). Despite maximal safe surgical resection followed by radiotherapy and temozolomide‐based chemotherapy, clinical outcomes remain poor, with most patients experiencing recurrence and limited long‐term survival (Weller et al. 2021). GBM is marked by diffuse invasion, rapid cellular adaptation, intratumoral heterogeneity, therapy resistance, and profound immune evasion. The limited benefit of immune checkpoint inhibitors in recurrent GBM, despite their efficacy in several extracranial solid tumors, highlights the distinctive immunological structure of the central nervous system (CNS) tumor microenvironment (TME) and the need to identify systemic and local modulators of antitumor immunity (Reardon et al. 2020).

The gut microbiota has emerged as a systemic regulator of immune homeostasis, inflammatory tone, and cancer treatment responsiveness, but its contribution to GBM remains incompletely defined and should be interpreted through a preclinical and early translational lens (Gopalakrishnan et al. 2018). The gut‐brain axis (GBA) represents a bidirectional communication network linking the gastrointestinal tract and CNS through immune, endocrine, metabolic, vascular, and neuronal pathways (Cryan et al. 2019). Within this framework, microbial metabolites such as short‐chain fatty acids (SCFAs), tryptophan‐derived indoles and kynurenine‐related metabolites, and polyamines may influence neuroinflammatory signaling, glial activity, blood‐brain barrier (BBB) function, and tumor‐associated immune responses (Cryan et al. 2019; Rothhammer et al. 2016). Recent metabolic studies from glioma models have further shown that GBM immune suppression is shaped not only by tumor‐cell metabolism but also by myeloid and microglial metabolic programs. Polyamine metabolism can support tumor‐associated myeloid cell survival under acidic stress, whereas microglia‐specific GLUT5‐dependent fructose metabolism may restrain inflammatory antigen‐presenting programs and adaptive antitumor immunity in the GBM microenvironment (Miska et al. 2021; Billingham et al. 2026).

Sequencing‐based and spatial studies have also reported microbial nucleic acid signatures and putative intratumoral bacterial signals in glioma and other solid tumor tissues (Li et al. 2024; Morad et al. 2025). These findings raise the possibility that tumor‐associated microbial signals may interact with immune and metabolic programs in GBM, although low microbial biomass, reagent contamination, sampling differences, and bioinformatic variability remain major limitations (Li et al. 2024; Morad et al. 2025; Eisenhofer et al. 2019). Therefore, claims regarding a functional intratumoral microbiome in GBM require careful validation using rigorous negative controls, orthogonal detection methods, and spatial localization approaches (Morad et al. 2025; Eisenhofer et al. 2019).

Experimental evidence further suggests that changes in gut microbial composition can influence glioma‐relevant immune biology. In antibiotic‐treated and fecal microbiota transplantation (FCT) mouse models, gut microbiome disruption altered Foxp3 expression in the glioma microenvironment and affected tumor progression, supporting a functional link between gut microbial ecology and glioma‐associated immune regulation (Fan et al. 2022). Preclinical studies of microbiota‐derived metabolites have extended this concept by showing that sodium butyrate can reduce glioma cell viability, promote apoptosis, alter cell‐cycle programs, and enhance anti‐PD‐1 efficacy in orthotopic glioma models, although these findings remain insufficient for direct clinical translation (Majchrzak‐Celińska et al. 2021; Li et al. 2025).

Substantial gaps remain in the field. Human evidence is still limited, causal mechanisms are incompletely mapped, and methodological heterogeneity complicates the interpretation of microbiome findings in low‐biomass CNS tumor samples (Morad et al. 2025; Eisenhofer et al. 2019). A cautious but biologically plausible model is emerging in which gut‐derived microbial signals, microbial metabolites, systemic immune modulation, and local metabolic programs within microglia and tumor‐associated myeloid cells (TAMCs) may converge on GBM neuroinflammation and treatment resistance (Cryan et al. 2019; Miska et al. 2021; Billingham et al. 2026). This review evaluates current evidence linking microbiota, neuroinflammatory signaling, and GBM biology, with emphasis on mechanistic plausibility, translational opportunities, and unresolved limitations.

## 2 Glioblastoma and the Immunological Tumor Microenvironment

The immunosuppressive TME of GBM facilitates immune evasion, tumor progression, and resistance to therapy. Although the CNS has historically been described as relatively immune‐privileged, this concept is now understood as context‐dependent rather than absolute (Sampson et al. 2020). Immune surveillance, meningeal lymphatic drainage, peripheral immune trafficking, and tumor‐driven inflammatory remodeling all contribute to the immunological activity of the GBM microenvironment (Sampson et al. 2020; Hu et al. 2020). Therefore, GBM should not be viewed as immunologically inert, but rather as an immune‐active tumor in which antitumor responses are redirected toward suppressive, dysfunctional, or metabolically constrained states (Jackson et al. 2019).

A defining feature of GBM immunity is the predominance of TAMCs, including brain‐resident microglia and bone marrow‐derived monocytes/macrophages (Andersen et al. 2021). These populations may account for approximately 30%–50% of the cellular tumor mass and are distributed across the tumor core, invasive margins, perivascular niches, and hypoxic regions (Andersen et al. 2021; Klemm et al. 2020). Rather than fitting a simple M1/M2 polarization model, GBM‐associated myeloid cells occupy heterogeneous transcriptional and functional states shaped by tumor genotype, hypoxia, acidity, cytokine exposure, and spatial localization within the tumor (Klemm et al. 2020; Ochocka et al. 2021). Glioma‐derived signals, including colony‐stimulating factor 1 (CSF‐1), CCL2, granulocyte‐macrophage colony‐stimulating factor, prostaglandins, and hypoxia‐related mediators, can promote suppressive myeloid programs that limit cytotoxic T‐cell activity and support invasion, angiogenesis, extracellular matrix remodeling, and therapy resistance (Andersen et al. 2021; Wang et al. 2022). These myeloid cells produce or respond to immunosuppressive mediators such as transforming growth factor‐beta (TGF‐β), interleukin‐10 (IL‐10), arginase‐1, and checkpoint‐related ligands, thereby weakening effective antitumor immunity (Andersen et al. 2021; Wang et al. 2022; Hu et al. 2025).

Recent work from the Miska/Chandel group has expanded this view by showing that metabolic adaptation within GBM‐associated myeloid cells is itself a mechanism of immune suppression. In murine glioma models, the arginine‐ornithine‐polyamine axis was enriched in TAMCs, allowing de novo production of polyamines such as putrescine and spermidine. Because polyamines are highly basic molecules, they buffer intracellular acidity and support TAMC survival and suppressive function within the acidic GBM TME. Pharmacological inhibition of polyamine synthesis with difluoromethylornithine reduced myeloid‐driven immunosuppression, increased CD8+ T‐cell‐associated antitumor activity, and improved responses to immune checkpoint blockade and radiotherapy in preclinical glioma models (Miska et al. 2021).

Microglia also contribute to GBM progression through specialized metabolic and immune programs that are not fully shared by infiltrating macrophages (Klemm et al. 2020; Ochocka et al. 2021). Billingham and colleagues identified microglia‐specific GLUT5/SLC2A5‐dependent fructose metabolism as another local metabolic mechanism that restrains antitumor immunity in GBM. In human GBM specimens and murine glioma models, GLUT5 expression was enriched in P2RY12+ microglia rather than CD163+ infiltrating macrophages or tumor cells. Loss of GLUT5 reduced tumor growth in CT‐2A, QPP4, and RCAS‐derived glioma models and generated a more inflammatory TME characterized by increased microglial antigen‐presentation markers, enhanced MHC‐I and MHC‐II expression, stronger IFN‐γ‐associated responses, increased CD8+ T‐cell activation, greater T‐cell clonal expansion, and increased B‐cell involvement. Mechanistically, microglial fructose uptake supported redox homeostasis through maintenance of the GSH/GSSG balance, reduced ROS‐associated inflammatory activation, suppressed IL‐1β responses, and decreased phagocytosis of apoptotic glioma cells, thereby limiting antigen‐presenting activity and adaptive antitumor immunity (Billingham et al. 2026). These findings indicate that the immunological phenotype of GBM is shaped not only by immune‐cell abundance but also by metabolic programs within resident and infiltrating myeloid populations (Miska et al. 2021; Billingham et al. 2026; Klemm et al. 2020; Ochocka et al. 2021).

Adaptive immune dysfunction further reinforces the suppressive GBM TME (Andersen et al. 2021). Regulatory T cells (Tregs) are enriched in GBM tissue and, in some studies, in the cerebrospinal fluid (CSF), where they suppress effector T‐cell activation and reduce antitumor immune responses (Li et al. 2026). Their recruitment and expansion are supported by chemokines such as CCL22 and by immunosuppressive cytokines within the tumor milieu, and increased Treg infiltration has been associated with poorer clinical outcomes in several glioma cohorts (Li et al. 2026). Chronic antigen exposure, hypoxia, nutrient limitation, adenosine accumulation, and suppressive myeloid signaling promote T‐cell dysfunction, with increased expression of inhibitory receptors such as PD‐1, TIM‐3, LAG‐3, and CTLA‐4 on tumor‐infiltrating lymphocytes (Collier 2018; Watowich and Gilbert 2023). In parallel, GBM cells and myeloid populations may express PD‐L1 and other inhibitory ligands, creating an immune checkpoint‐rich environment that contributes to the limited efficacy of checkpoint blockade in unselected GBM populations (Andersen et al. 2021; Collier 2018).

Immune dysfunction in GBM is not restricted to the local tumor site (Andersen et al. 2021). Systemic immunosuppression, including lymphopenia, corticosteroid exposure, impaired T‐cell function, and sequestration of T cells in the bone marrow, further limits antitumor immunity and may reduce responsiveness to immunotherapeutic approaches (Chongsathidkiet et al. 2018; Stepanenko et al. 2024). This local and systemic immunosuppressive network provides a major barrier to durable immune control of GBM and underscores the importance of identifying upstream regulators that influence immune‐cell recruitment, polarization, metabolism, and effector function (Andersen et al. 2021; Stepanenko et al. 2024). Gut microbiota‐derived inflammatory signals and metabolites represent one potential upstream layer because they can influence microglial maturation, peripheral cytokine tone, and myeloid‐cell programming, although the extent to which these mechanisms directly operate in patients with GBM remains insufficiently established (Thion et al. 2018; Morais et al. 2021). Understanding how microbiota‐associated immune signaling intersects with the GBM TME may help clarify why antitumor immune responses remain weak despite the presence of an immunologically active tumor niche (Andersen et al. 2021; Morais et al. 2021).

## 3 Gut–Brain Axis and Microbial Modulation of Neuroinflammation

The GBA is a dynamic bidirectional communication system between the gastrointestinal tract and the CNS, integrating immune, metabolic, endocrine, vascular, and neuronal pathways (Fung et al. 2017; D'Alessandro et al. 2021). The gut microbiota, which includes bacteria, archaea, fungi, viruses, and their metabolic products, contributes to immune education, barrier regulation, neurodevelopmental processes, and glial homeostasis (Fung et al. 2017; Abdel‐Haq et al. 2019). In the context of GBM, the GBA is best interpreted as a biologically plausible regulatory framework rather than a fully established causal pathway because most mechanistic evidence is derived from preclinical neuroinflammatory models, glioma‐bearing mice, or association‐based human studies (D'Alessandro et al. 2021; Zhang et al. 2022).

One of the most relevant mechanisms involves microbiota‐derived SCFAs, including acetate, propionate, and butyrate (Dalile et al. 2019). Rather than simply breaching the BBB, SCFAs can influence BBB integrity, endothelial tight‐junction regulation, and immune signaling at the neurovascular interface (Fang et al. 2019; Hoyles et al. 2018). Germ‐free mice show increased BBB permeability, whereas colonization with a conventional microbiota or exposure to SCFA‐producing bacteria can reduce BBB permeability and support barrier maturation (Fang et al. 2019). SCFAs also participate in microglial development and functional maturation (Thion et al. 2018; Abdel‐Haq et al. 2019). Erny et al. (2015)showed that germ‐free mice display immature and functionally altered microglia, and that microbiota recolonization or SCFA supplementation can partly restore microglial defects. These findings are not GBM‐specific, but they provide a mechanistic basis for considering how microbiota‐derived metabolites may shape microglial states within glioma‐relevant inflammatory contexts (D'Alessandro et al. 2021; Zhang et al. 2022).

Microbiota‐dependent regulation of CNS inflammation also involves tryptophan metabolism. Commensal microorganisms can convert dietary tryptophan into indole derivatives that activate aryl hydrocarbon receptor (AhR) signaling in astrocytes, microglia, and immune cells (Roager and Licht 2018). In experimental neuroinflammatory models, microbial tryptophan metabolites acting through AhR pathways can limit pathogenic glial activation and modulate CNS inflammation (Roager and Licht 2018). In GBM, this pathway remains mechanistically plausible because AhR signaling intersects with immune tolerance, myeloid‐cell function, and tumor‐associated inflammation, but direct GBM‐specific evidence is still limited (Zhang et al. 2022; Zahariah and Midi 2023). The kynurenine branch of tryptophan metabolism is also relevant to glioma biology because it can promote immunosuppressive signaling, T‐cell dysfunction, and tumor‐supportive metabolic adaptation (Zahariah and Midi 2023; Jacquerie et al. 2024).

A second layer of communication occurs through peripheral immune priming. Gut dysbiosis can alter systemic inflammatory tone, circulating microbial products, monocyte programming, and cytokine profiles, which may influence immune‐cell trafficking and function within the CNS (Fung et al. 2017; Dalile et al. 2019). In glioma models, gut microbiome disturbance has been associated with altered Foxp3 expression in the TME and changes in tumor growth, suggesting that intestinal microbial ecology can affect glioma‐associated immune regulation in vivo (Fan et al. 2022). These findings support a link between gut microbiota and glioma immunity, but they should not be interpreted as proof that dysbiosis directly drives human GBM initiation (Fan et al. 2022; Zhang et al. 2022).

Neuronal and neuroendocrine pathways may also participate in GBA signaling. Microbial products and gut‐derived neuroactive molecules can influence vagal afferent signaling and hypothalamic‐pituitary‐adrenal (HPA) axis activity, thereby shaping systemic stress responses and inflammatory tone (D'Alessandro et al. 2021; Bonaz et al. 2018). However, evidence connecting vagal signaling directly to GBM‐associated edema, seizure thresholds, or tumor progression remains indirect; therefore, this mechanism should be considered a broader neuroimmune route rather than a validated GBM‐specific pathway (D'Alessandro et al. 2021; Bonaz et al. 2018; Khabibov et al. 2022).

Human genetic and association‐based studies provide additional, but still preliminary, support for a relationship between gut microbiota composition and GBM susceptibility. Mendelian randomization analyses have reported associations between selected microbial taxa and GBM risk (Wang et al. 2023; Pleguezuelos et al. 2023). For example, Ruminococcaceae and Faecalibacterium have been associated with reduced GBM risk in some datasets, whereas Peptostreptococcaceae and the Eubacterium brachy group have been associated with increased susceptibility (Wang et al. 2023; Pleguezuelos et al. 2023; Nejadrostam et al. 2026). Because Mendelian randomization identifies genetically predicted associations rather than direct biological mechanisms, these findings should be treated as hypothesis‐generating and require validation through longitudinal cohorts, metagenomics, metabolomics, and mechanistic glioma models (Wang et al. 2023; Pleguezuelos et al. 2023). Nevertheless, the depletion of SCFA‐producing commensals and enrichment of pro‐inflammatory microbial patterns may represent one route through which microbial ecology could influence systemic immune tone and glioma‐relevant neuroinflammation (Dalile et al. 2019; Wang et al. 2023; Pleguezuelos et al. 2023).

**FIGURE 1:**
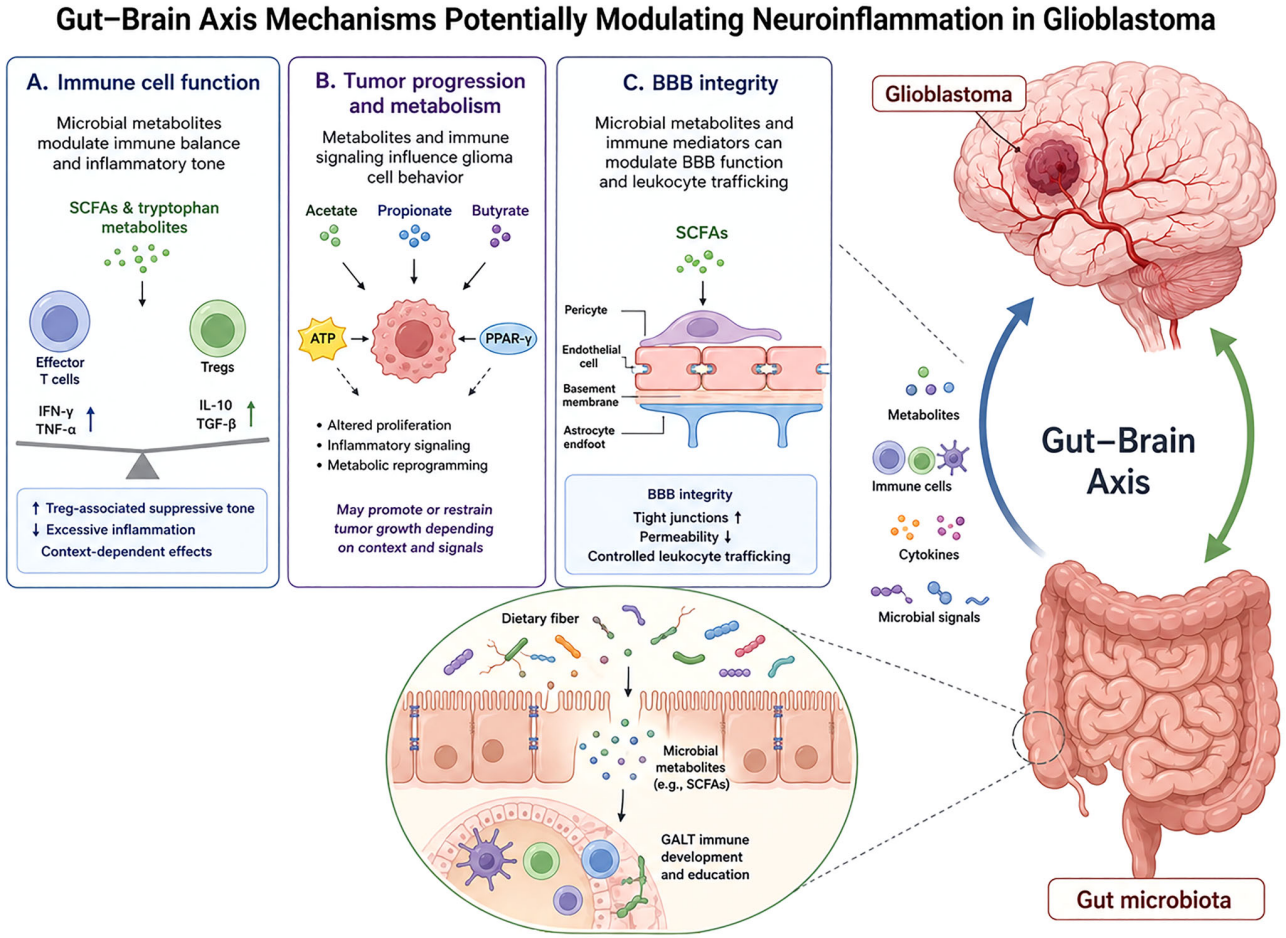
Gut–brain axis mechanisms in glioblastoma‐associated neuroinflammation. The figure summarizes how gut microbiota may influence glioblastoma biology through immune‐cell modulation, microbial metabolites, blood–brain barrier regulation, cytokine signaling, and microbial signals. These pathways may affect effector T cells, Tregs, glioma cell behavior, BBB integrity, and the inflammatory tumor microenvironment. SCFAs, short‐chain fatty acids; GALT, gut‐associated lymphoid tissue; BBB, blood–brain barrier; Tregs, regulatory T cells; IL‐10, interleukin‐10; TGF‐β, transforming growth factor‐beta; IFN‐γ, interferon‐gamma; TNF‐α, tumor necrosis factor‐alpha.

Collectively, current evidence supports the gut microbiota as a potential regulator of neuroimmune processes relevant to GBM, particularly through SCFA‐mediated microglial regulation, BBB modulation, tryptophan‐AhR signaling, peripheral immune priming, and systemic inflammatory tone (Fung et al. 2017; D'Alessandro et al. 2021; Dalile et al. 2019; Roager and Licht 2018). The therapeutic implications of these mechanisms remain experimental, and microbiota‐targeted strategies should be framed as investigational adjuvant approaches rather than established GBM treatments (D'Alessandro et al. 2021; Zhang et al. 2022). The major GBA mechanisms potentially connecting gut microbial ecology to GBM‐associated neuroinflammation are summarized in Table 1 and Figure 1.

**TABLE 1:** Gut‐brain axis mechanisms potentially modulating neuroinflammation in glioblastoma.

Mechanistic pathway	Microbial mediators or signals	Principal neural or immune targets	GBM‐relevant biological interpretation	Translational implications	Key evidence	Ref
SCFA‐microglia regulation	Acetate, propionate, butyrate	Microglia, SCFA‐sensitive signaling pathways, epigenetic regulators	Microbiota‐derived SCFAs support microglial maturation and may shape microglial inflammatory states relevant to glioma immunity	SCFA‐producing diets, prebiotics, or metabolite‐based adjuvant strategies remain investigational	Preclinical neuroimmunology evidence from germ‐free and SCFA‐supplemented mice	(Thion et al. 2018; Abdel‐Haq et al. 2019; Dalile et al. 2019)
BBB and neurovascular modulation	SCFAs, microbial metabolites, microbial‐associated inflammatory products	Brain endothelial cells, tight junction proteins, astrocyte‐endothelial interface	Gut microbiota can influence BBB integrity; extrapolation to the heterogeneous GBM blood‐tumor barrier requires caution	Barrier‐protective microbiome strategies require validation in glioma models	Germ‐free and colonization studies showing microbiota‐dependent BBB regulation	(Dalile et al. 2019; Hoyles et al. 2018; Parker et al. 2020)
Tryptophan‐AhR signaling	Indole derivatives, kynurenine‐related metabolites	AhR signaling in astrocytes, microglia, T cells, and myeloid cells	Microbial tryptophan metabolites regulate CNS inflammation through AhR‐dependent pathways; GBM‐specific consequences remain incompletely defined	AhR pathway modulation and restoration of indole‐producing commensals are experimental concepts	Preclinical CNS inflammation studies and glioma‐relevant immunometabolic rationale	(Rothhammer et al. 2016; Roager and Licht 2018)
Peripheral immune priming	Dysbiosis‐associated microbial products, LPS‐related signaling, circulating metabolites	Monocytes, T cells, bone marrow‐derived immune cells, cytokine networks	Gut microbial imbalance may alter systemic inflammatory tone and immune‐cell programming before CNS infiltration	Microbial consortia or immune‐metabolic adjuvants could be explored with checkpoint or myeloid‐targeted therapies	Preclinical glioma models and broader cancer immunology evidence	(Fan et al. 2022; D'Alessandro et al. 2020)
Vagal and neuroendocrine signaling	Microbial neuroactive compounds, gut‐derived inflammatory signals	Vagus nerve, HPA axis, systemic stress and inflammatory pathways	May influence neuroimmune tone, but direct evidence in GBM progression, edema, or seizure biology remains indirect	Psychobiotics, stress‐axis modulation, and vagal approaches remain speculative in GBM	General GBA evidence, limited GBM‐specific validation	(Morais et al. 2021; Bonaz et al. 2018)
Microbiota composition and predicted GBM susceptibility	Ruminococcaceae, *Faecalibacterium*, Peptostreptococcaceae, *Eubacterium brachy* group	Systemic immunity, glial inflammatory tone, microbial metabolite networks	Genetically predicted microbial taxa have been associated with GBM risk, but biological causality remains unproven	Requires longitudinal human cohorts and paired metagenomic‐metabolomic validation	Mendelian randomization and association‐based human evidence	(Wang et al. 2023; Pleguezuelos et al. 2023)
Microbiome manipulation in glioma models	Antibiotic‐induced dysbiosis, fecal microbiota transplantation, altered microbial ecology	Foxp3+ regulatory programs, innate immune cells, glioma immune microenvironment	Experimental microbiome perturbation can modify glioma growth and immune markers in mice	Microbiota‐targeted interventions remain preclinical and should not be presented as established therapy	Mouse glioma studies using microbiome perturbation	(D'Alessandro et al. 2020; Green et al. 2025)

## 4 Intratumoral Microbiota: Bacterial and Fungal Signatures in Glioblastoma

Recent studies have renewed interest in the possibility that microbial signals may be detectable within brain tumors, including glioma and GBM (Li et al. 2024; Morad et al. 2025). Rather than overturning the concept of CNS immune protection or proving stable microbial colonization, current evidence should be interpreted as support for low‐abundance bacterial and fungal nucleic acid signatures, bacterial components, and spatially restricted microbial signals in selected brain tumor specimens (Li et al. 2024; Morad et al. 2025; Dubiński et al. 2026). This distinction is important because brain tumors are low‐biomass samples, and microbial readouts are highly vulnerable to contamination, batch effects, sampling conditions, and bioinformatic artefacts (Eisenhofer et al. 2019; Fierer et al. 2025).

Earlier pan‐cancer sequencing analyses of microbial reads from large cancer datasets stimulated interest in tumor‐associated microbial signatures, but one influential TCGA‐based report was later retracted because of concerns regarding the robustness of cancer‐associated microbial signatures (Poore et al. 2024). Therefore, retracted database‐mining evidence should not be used as primary support for intratumoral microbiota in GBM (Poore et al. 2024). More recent glioma‐focused studies have used multi‐omics, immunohistochemistry, multicolor immunofluorescence, fluorescence in situ hybridization, and spatial approaches to strengthen the evidence base (Li et al. 2024; Morad et al. 2025). A multi‐omics analysis of paired glioma and adjacent normal brain tissues reported enrichment of genera such as Fusobacterium, Longibaculum, Intestinimonas, Pasteurella, Limosilactobacillus, and Arthrobacter in glioma tissues, together with bacterial RNA and lipopolysaccharide signals (Li et al. 2024). In experimental validation, Fusobacterium nucleatum promoted glioma cell proliferation and increased CCL2, CXCL1, and CXCL2 expression in glioma models. These findings suggest that at least some bacterial signals may be biologically relevant, although their frequency, viability, spatial distribution, and patient‐level generalizability remain unresolved (Li et al. 2024; Morad et al. 2025; Dubiński et al. 2026).

Independent brain tumor studies using high‐resolution imaging and sequencing have also detected intracellular bacterial elements, including bacterial RNA, DNA, and membrane‐associated signals, in glioma and brain metastasis specimens (Morad et al. 2025). These studies reported distinct bacterial signatures in tumor tissue compared with non‐cancerous brain tissue and showed that only a subset of low‐biomass sequencing signals remained after stringent contaminant filtration, emphasizing the need for orthogonal validation. The fact that many samples lose detectable signal after filtration does not exclude biological microbial signals, but it does show that taxonomic conclusions from low‐biomass sequencing alone should be treated cautiously (Morad et al. 2025; Eisenhofer et al. 2019).

Several non‐exclusive mechanisms may explain how microbial signals could appear in GBM tissue. Bacterial products or microbial fragments may enter through a disrupted blood‐brain or blood‐tumor barrier, particularly in regions with abnormal vascular permeability (Morad et al. 2025; Nejman et al. 2020). Alternatively, microbial material may be transported by immune cells, including macrophages or dendritic cells, or may accumulate in necrotic, hypoxic, or poorly perfused tumor niches that favor persistence of microbial components (Morad et al. 2025; Sarkaria et al. 2018). A more cautious interpretation is that GBM‐associated microbial signals may reflect a mixture of intracellular bacterial elements, non‐cultivable bacterial components, immune‐cell‐associated microbial material, and potential contamination, rather than uniform colonization by viable organisms (Morad et al. 2025; Eisenhofer et al. 2019). Their functional relevance should therefore be evaluated using spatial localization, matched negative controls, reagent controls, culture‐independent validation, and mechanistic models (Morad et al. 2025; Fierer et al. 2025; Meng et al. 2023).

Fungal signatures have also been investigated in cancer tissues. Pan‐cancer mycobiome studies have reported fungal DNA and fungal cells at low abundance across multiple cancer types, with cancer‐type‐specific fungal ecologies and interactions with bacterial communities (Narunsky‐Haziza et al. 2022; Dohlman et al. 2022). For GBM, however, evidence for a functional mycobiome remains much weaker than evidence for bacterial signals (Morad et al. 2025; Narunsky‐Haziza et al. 2022). Fungal genera such as Candida and Malassezia should be discussed as putative low‐abundance signatures rather than established GBM‐resident communities (Narunsky‐Haziza et al. 2022). This caution is particularly important because Malassezia and other skin‐associated fungi are common contaminants in low‐biomass sequencing workflows (Eisenhofer et al. 2019; Fierer et al. 2025).

Contamination control is central to this field. Reliable identification of intratumoral microbial signals requires blank extraction controls, reagent controls, environmental controls, batch‐aware bioinformatic filtering, dual‐index sequencing strategies, and orthogonal spatial methods such as FISH, immunostaining, spatial molecular imaging, or digital spatial profiling (Morad et al. 2025; Eisenhofer et al. 2019; Fierer et al. 2025). When possible, paired tumor, adjacent normal brain, blood, stool, oral, and skin‐control samples should be included to distinguish tumor‐enriched signals from procedural or anatomical background (Morad et al. 2025; Fierer et al. 2025; Wang et al. 2025).

At present, intratumoral microbiota in GBM should be framed as an emerging and technically challenging field. Available evidence supports the presence of low‐abundance microbial signals in at least a subset of glioma specimens, but it does not yet establish a universal, viable, or clinically actionable GBM microbiome (Li et al. 2024; Morad et al. 2025; Eisenhofer et al. 2019; Dohlman et al. 2022). The most defensible interpretation is that microbial signals may interact with immune tone, cytokine signaling, antigen presentation, metabolic stress, or treatment response in selected contexts, while rigorous validation is still required before these findings can be translated into diagnostic or therapeutic strategies (Li et al. 2024; Morad et al. 2025; Fierer et al. 2025).

## 5 Microbial Metabolites and Epigenetic Regulation in GBM Progression

Microbial metabolites are important mediators of host‐microbe communication and can influence immune regulation, epithelial and endothelial barrier function, transcriptional programs, and cellular metabolism (Krautkramer et al. 2016). In GBM, the most relevant metabolite classes include SCFAs, tryptophan‐derived metabolites, and polyamines, although their mechanistic importance differs across experimental systems and levels of evidence (Miska et al. 2021; Liu et al. 2023; Li et al. 2022). These metabolites should be considered microbiota‐associated or host‐microbe‐intersecting signals rather than uniformly gut‐derived molecules because several pathways, especially kynurenine and polyamine metabolism, are also strongly regulated by host and tumor cells (Miska et al. 2021; Li et al. 2022).

SCFAs are produced mainly through anaerobic fermentation of dietary fibers by intestinal bacteria, including SCFA‐producing taxa such as Faecalibacterium prausnitzii and Roseburia species (Dalile et al. 2019). Butyrate, propionate, and acetate can regulate immune and metabolic signaling, with butyrate and propionate acting as histone deacetylase (HDAC) inhibitors in several cellular contexts (Liu et al. 2023; Li et al. 2022). Through HDAC inhibition, SCFAs may affect chromatin accessibility, inflammatory gene expression, microglial maturation, regulatory T‐cell differentiation, and tumor‐cell survival programs (Liu et al. 2023; Li et al. 2022). However, their effects are context‐dependent: SCFAs may support anti‐inflammatory immune regulation in some settings while exerting direct cytotoxic or differentiation‐modulating effects in selected tumor cell models (Liu et al. 2023; Corrêa‐Oliveira et al. 2016).

The evidence for butyrate in neural and glial tumor models has become more specific. In SH‐SY5Y human neuroblastoma cells, sodium butyrate reduced cell viability in a dose‐ and time‐dependent manner, inhibited colony formation, and induced apoptosis with nuclear condensation and fragmentation; treatment with 10 mM sodium butyrate for 48 h produced an apoptosis rate of approximately 22.2% (Kaya Sezginer et al. 2025). This supports the broader pro‐apoptotic potential of butyrate in neural tumor cell models, but it should not be treated as GBM‐specific evidence (Kaya Sezginer et al. 2025; Getachew et al. 2021). Direct GBM‐relevant evidence is available, but it remains largely preclinical. In human GBM cell lines A‐172, T98G, and U‐138 MG, sodium butyrate reduced viability in a dose‐ and cell‐line‐dependent manner, induced apoptosis, altered cell‐cycle distribution, increased reactive oxygen species in some cell‐line contexts, upregulated Wnt/β‐catenin pathway antagonists such as SFRP1 and RUNX3, and down regulated the epigenetic regulator UHRF1 (Majchrzak‐Celińska et al. 2021). The same study showed that sodium butyrate combined with curcuminoids produced stronger cytotoxicity and G2/M arrest, although these findings were based on in vitro GBM cell‐line systems and require validation in patient‐derived and in vivo models (Majchrzak‐Celińska et al. 2021; Shahani et al. 2025).

A more recent glioma study extended these observations to immune therapy. In U251 and GL261 glioma models, sodium butyrate reduced cell viability, increased Annexin V‐positive apoptosis, reduced survivin/BIRC5 expression, induced G2/M cell‐cycle arrest, and down regulated CDC2, CDC25C, CCNA2, and CCNB1. Sodium butyrate also reduced class I HDAC proteins, particularly HDAC1, HDAC2, and HDAC3, while increasing PD‐L1 expression through PI3K/AKT signaling in U251 cells. In an orthotopic GL261 model, sodium butyrate alone showed limited in vivo antitumor activity, but its combination with anti‐PD‐1 therapy reduced tumor burden, increased CD4+ and CD8+ T‐cell infiltration, enhanced IFN‐γ production by tumor‐infiltrating lymphocytes, and prolonged median survival compared with either treatment alone. These data suggest that butyrate may influence both glioma‐intrinsic epigenetic programs and antitumor immune responsiveness, but the evidence remains preclinical and should not be presented as clinical proof of efficacy in GBM patients (Li et al. 2025).

Tryptophan metabolism represents another important host‐microbiota interface. Microbial indole derivatives can activate AhR signaling in astrocytes, microglia, and immune cells, thereby modulating neuroinflammatory responses in experimental CNS models (Rothhammer et al. 2016; Roager and Licht 2018). In glioma, tryptophan metabolism is also shaped by tumor and immune‐cell pathways, including indoleamine 2,3‐dioxygenase‐mediated conversion of tryptophan into kynurenine metabolites (Zahariah and Midi 2023; Li et al. 2022). These metabolites may promote immune tolerance, regulatory T‐cell expansion, myeloid‐cell dysfunction, and reduced effector T‐cell activity within the GBM microenvironment (Zahariah and Midi 2023; Li et al. 2022; Medikonda et al. 2025). The microbial contribution to this pathway in GBM remains less clearly defined than the tumor‐cell and immune‐cell contribution, so microbial indole and kynurenine‐related mechanisms should be described as plausible modulators rather than established drivers of GBM progression (Zahariah and Midi 2023; Li et al. 2022; Toumazi et al. 2026).

Polyamines, including putrescine, spermidine, and spermine, form a separate class of metabolites relevant to GBM. They are produced through host metabolic pathways and can also be influenced by microbial metabolism and diet (Sagar et al. 2021; Tofalo et al. 2019). Polyamines regulate cell proliferation, chromatin structure, oxidative stress responses, DNA stability, and immune‐cell function (Sagar et al. 2021; Nakamura et al. 2021). In GBM, polyamine metabolism has particular importance because it links metabolic adaptation to myeloid‐cell immunosuppression (Miska et al. 2021). The arginine‐ornithine‐polyamine axis can support tumor‐associated myeloid cell survival in the acidic GBM microenvironment by buffering intracellular pH, thereby preserving suppressive myeloid function (Miska et al. 2021; Holbert et al. 2024). Because this mechanism was already demonstrated in preclinical glioma models, polyamines should be discussed as host‐microbe‐intersecting metabolites with both tumor‐cell and immune‐cell relevance, not as purely microbial products (Miska et al. 2021; Tofalo et al. 2019).

**FIGURE 2:**
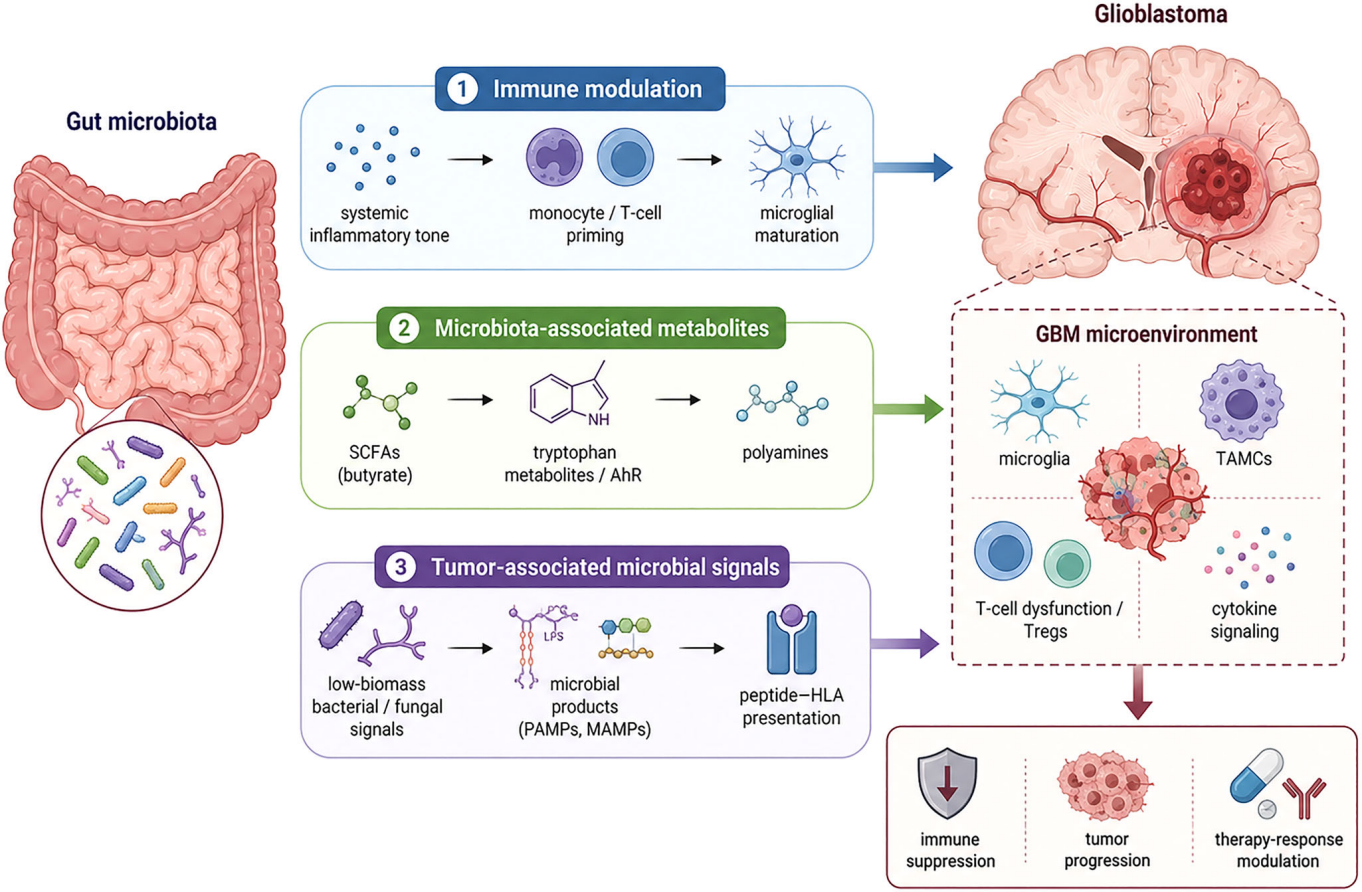
Gut microbiota–GBM communication pathways. The figure summarizes three potential routes by which gut microbiota may influence glioblastoma‐associated neuroinflammation: immune modulation, microbiota‐associated metabolites, and tumor‐associated microbial signals. These mechanisms may reshape the GBM microenvironment by affecting microglia, TAMCs, T‐cell dysfunction, cytokine signaling, and peptide–HLA presentation, with possible consequences for immune suppression, tumor progression, and therapy response.

Microbial and host‐intersecting metabolites therefore provide a mechanistic bridge between diet, intestinal ecology, systemic immune tone, glial function, tumor‐cell epigenetics, and therapy response (Dalile et al. 2019; Krautkramer et al. 2016). The strongest GBM‐relevant evidence currently supports preclinical roles for sodium butyrate and polyamine metabolism, whereas tryptophan‐AhR signaling remains biologically plausible but less directly validated in GBM‐specific microbiome studies (Miska et al. 2021; Li et al. 2025). Future work should integrate metagenomics, metabolomics, spatial transcriptomics, patient‐derived glioma models, and controlled intervention studies to determine whether these metabolites are causal contributors, adaptive byproducts, or therapeutic vulnerabilities in GBM progression. The potential convergence of microbiota‐associated metabolites, immune modulation, and microbial antigen signals within the GBM microenvironment is summarized in Figure 2.

## 6 Microbiota‐Derived Peptides and HLA Cross‐Reactivity in Brain Tumors

Molecular mimicry provides a potential mechanism through which microbial antigens may influence antitumor immunity. In this process, microbial peptides share sequence or structural similarity with tumor‐associated antigens or neoepitopes and may be presented by major histocompatibility complex (MHC) molecules, thereby shaping T‐cell recognition (Tagliamonte et al. 2023; Zhu et al. 2022). In cancer biology, this concept gained support from immunopeptidomic studies in melanoma showing that bacteria‐derived peptides can be presented on HLA‐I and HLA‐II molecules and may elicit immune reactivity (Kalaora et al. 2021). These findings established a broader principle that intratumoral or tumor‐associated bacteria can contribute to the antigenic landscape of malignant tissues (Kalaora et al. 2021; Zhang et al. 2024).

GBM‐specific evidence has now extended this concept to brain tumors. Naghavian et al. demonstrated that HLA molecules from GBM tissues and GBM cell lines can present bacteria‐specific peptides. Using immunopeptidomics, the authors identified HLA class II‐restricted bacterial peptides in GBM samples and showed that selected microbial peptides could activate tumor‐infiltrating lymphocytes ex vivo (Naghavian et al. 2023). This evidence suggests that bacterial antigens may contribute to the immunopeptidome of GBM and could influence local T‐cell recognition. However, the source of these peptides, whether derived from viable intratumoral bacteria, intracellular bacterial components, phagocytosed microbial material, or low‐abundance microbial remnants, remains an important methodological and biological question.

Microbial peptide presentation may have divergent consequences in GBM. On one hand, bacterial peptides that resemble tumor antigens could broaden antitumor T‐cell repertoires and support immune recognition of malignant cells (Tagliamonte et al. 2023; Naghavian et al. 2023). On the other hand, chronic or low‐affinity stimulation may contribute to immune dysfunction, tolerance, or exhaustion, particularly within the metabolically constrained and myeloid‐rich GBM microenvironment (Collier 2018). The biological outcome is therefore likely to depend on HLA genotype, peptide abundance, antigen‐presenting cell state, T‐cell receptor affinity, spatial localization of microbial signals, and the suppressive context of the TME (Watowich and Gilbert 2023; Taylor et al. 2021).

This mechanism also has implications for microbiota‐informed immunotherapy. If microbial peptides contribute to GBM antigenicity, changes in gut or tumor‐associated microbial communities could theoretically alter the pool of cross‐reactive antigens available for T‐cell priming (Zhang et al. 2024; Naghavian et al. 2023). This possibility provides a mechanistic rationale for integrating microbial antigen discovery with HLA typing, immunopeptidomics, single‐cell T‐cell receptor sequencing, and neoantigen prediction in GBM research (Taylor et al. 2021). At the same time, this field remains early and should not be interpreted as evidence that probiotics, antibiotics, or dietary interventions can reliably reshape the GBM T‐cell repertoire in patients (Zhang et al. 2024; Naghavian et al. 2023; Tian and Ma 2023).

The interaction between microbial antigens and HLA presentation may support future vaccine design, microbial‐peptide‐based antigen discovery, and personalized immunotherapy strategies in GBM (Tagliamonte et al. 2023; Naghavian et al. 2023; Taylor et al. 2021). The most immediate translational value is likely to come from identifying reproducible bacterial peptide‐HLA complexes, determining whether they activate functional antitumor T cells, and establishing whether these responses can be safely amplified without promoting tolerance or off‐target inflammation (Taylor et al. 2021; Hill et al. 2025).

## 7 Preclinical Models and Experimental Manipulation of the Microbiome

Preclinical models have been central to testing whether gut microbiota can influence glioma growth, immune regulation, and treatment responsiveness under controlled conditions (Fan et al. 2022; D'Alessandro et al. 2020). Antibiotic‐treated rodents, FMT, diet‐based interventions, metabolite supplementation, and humanized microbiome mouse models have each provided mechanistic insight into the gut‐immune‐brain interface in glioma (Fan et al. 2022; D'Alessandro et al. 2020; Green et al. 2025). These models are particularly useful because they allow microbial communities to be manipulated before or during tumor development, while immune infiltration, cytokine expression, tumor growth, and therapeutic response can be measured in parallel (Fan et al. 2022; D'Alessandro et al. 2020; Chevalier et al. 2025).

Fan et al. provided one of the clearest experimental demonstrations that gut microbiome disturbance can affect glioma progression. In a glioma‐bearing mouse model, antibiotic‐induced microbiome depletion promoted tumor growth and was associated with reduced Foxp3 expression in brain tissue, whereas FMT partially restored microbial balance, increased Foxp3 expression, and slowed tumor progression. These findings support a functional relationship between gut microbial ecology and glioma‐associated immune regulation, but they should be interpreted within the limits of mouse glioma models and should not be considered direct clinical evidence for FMT in GBM patients (Fan et al. 2022).

Dietary and nutrient‐based models have also contributed to this field. Kim et al. reported that supplementation with a high‐glucose drink enhanced antitumor immune responses in GBM‐bearing mice through gut microbiota modulation. The intervention increased CD8+ T‐cell‐mediated antitumor immunity, and its beneficial effect was reduced when the microbiota was depleted, indicating that the antitumor response depended at least partly on microbial composition. This finding is mechanistically important because it links diet, gut microbial ecology, and antitumor immunity; however, it should not be generalized into a dietary recommendation for patients with GBM (Kim et al. 2023).

Other dietary exposure models indicate that microbiota changes do not always translate into measurable tumor‐volume effects. A metagenomic and transcriptomic analysis of aspartame exposure in a mouse GBM model showed that aspartame altered gut microbial composition, including changes in Rikenellaceae abundance, and modified tumor‐associated gene‐expression programs, but it did not significantly change tumor growth. This result is useful because it shows that microbiome perturbation may affect molecular programs without producing an immediate change in tumor size, highlighting the need to evaluate immune, transcriptomic, metabolomic, and survival endpoints rather than tumor volume alone (Meng et al. 2025).

Humanized microbiome models are beginning to bridge the gap between mouse microbiota studies and patient‐level relevance. Green et al. showed that human gut microbial communities transplanted into glioma‐bearing mice influenced immune‐cell composition and gene activation patterns within brain tumors, both under control conditions and during immune checkpoint blockade (Green et al. 2025). Similarly, a humanized microbiome mouse model using fecal microbiota from patients with high‐grade glioma found that patient‐derived gut microbiota could promote glioma progression, potentially through metabolite‐linked mechanisms involving sphingosine 1‐phosphate (Wang et al. 2025). These studies suggest that patient‐derived microbial communities can modify glioma biology in vivo, although donor variability, engraftment efficiency, housing conditions, diet, and mouse strain effects remain major sources of experimental variability (Green et al. 2025; Wang et al. 2025; Dees et al. 2021).

Microbial metabolite interventions have added another experimental layer. Sodium butyrate has been tested in glioma cell models and orthotopic glioma‐bearing mice, where it suppressed glioma cell growth in vitro and enhanced anti‐PD‐1 efficacy in vivo by increasing CD4+ and CD8+ T‐cell infiltration and IFN‐γ production (Li et al. 2025). Because butyrate has both epigenetic and immunomodulatory properties, these findings support the concept that microbiota‐derived metabolites may function as immune‐adjuvant signals in glioma models. Their translation to human GBM requires careful pharmacokinetic, safety, dosing, and patient‐stratification studies (Li et al. 2025; Haddad et al. 2021; Gaskarth et al. 2025).

Despite their value, preclinical microbiome models have important limitations. The immune systems, microbial ecology, diet, metabolism, and barrier physiology of mice differ substantially from those of humans (Haddad et al. 2021). Orthotopic implantation models do not fully reproduce the genetic evolution, spatial heterogeneity, necrosis, treatment exposure, and long‐term immune adaptation seen in human GBM (Gómez‐Oliva et al. 2021). Antibiotic cocktails can also have microbiota‐independent effects on host metabolism and immunity, while FMT can introduce donor‐specific and facility‐dependent variability (Fan et al. 2022; Wang et al. 2025). Future translational studies should integrate patient‐derived glioma models, spontaneous or genetically engineered glioma systems, controlled gnotobiotic approaches, longitudinal microbiome‐metabolome profiling, and immune phenotyping to determine whether microbiome‐targeted strategies can reproducibly modify GBM progression or therapeutic response (Haddad et al. 2021; Gómez‐Oliva et al. 2021).

## 8 Clinical Implications, Translational Barriers, and Future Directions

The emerging links among gut microbiota, neuroinflammation, tumor‐associated microbial signals, and GBM immunity have several potential clinical implications, but their current translational status remains preliminary (Zhang et al. 2022). At present, microbiome‐related findings in GBM should be viewed as hypothesis‐generating and mechanistically informative rather than ready for routine diagnostic or therapeutic use (Zhang et al. 2022; THERApeutic Outcomes Related to Gut microBIOME in Glioblastoma (GBM)). The most clinically relevant opportunities include microbiome‐informed biomarkers, immune‐metabolic patient stratification, adjuvant modulation of antitumor immunity, and identification of microbial or microbial‐like antigens that may expand the repertoire of targetable GBM‐associated epitopes (Zhang et al. 2024; Lei et al. 2025).

Microbiome‐based biomarkers are an attractive but still immature concept in GBM. Human association studies and Mendelian randomization analyses have linked selected microbial taxa with GBM risk, including inverse associations for some short‐chain‐fatty‐acid‐producing taxa such as Ruminococcaceae and Faecalibacterium, and positive associations for taxa such as Peptostreptococcaceae and the Eubacterium brachy group (Wang et al. 2023; Pleguezuelos et al. 2023). These associations should not be interpreted as validated diagnostic biomarkers because they do not establish direct biological causality, and may be influenced by host genetics, geography, diet, medication exposure, antibiotic use, tumor stage, and statistical model assumptions (Dubiński et al. 2026; Lei et al. 2025). Future biomarker development will require longitudinal stool, blood, tumor, and CSF sampling combined with metagenomics, metabolomics, immune profiling, and treatment‐response data (THERApeutic Outcomes Related to Gut microBIOME in Glioblastoma (GBM)).

Tumor‐associated microbial signatures may also contribute to risk classification or immunophenotyping, but this area requires particular caution (Morad et al. 2025; Li et al. 2025). Brain tumors are low‐biomass specimens, and microbial signals detected in tumor tissue can be affected by reagent contamination, skin‐associated organisms, surgical sampling conditions, batch effects, and bioinformatic filtering strategies (Eisenhofer et al. 2019; Fierer et al. 2025). Therefore, microbial DNA, RNA, lipopolysaccharide signals, or spatial bacterial markers should be interpreted only when supported by adequate negative controls, paired tissue comparisons, orthogonal validation, and spatial localization methods (Morad et al. 2025; Fierer et al. 2025; Li et al. 2025). These safeguards are especially important if microbial signatures are proposed as diagnostic markers, prognostic indicators, or therapeutic targets (Eisenhofer et al. 2019; Fierer et al. 2025).

Microbiota‐targeted interventions also remain investigational in GBM. Preclinical studies suggest that antibiotic‐induced dysbiosis, FMT, diet‐based modulation, humanized microbiome models, and microbial metabolites can influence glioma‐associated immune responses and tumor behavior (Fan et al. 2022; Green et al. 2025; Wang et al. 2025). However, these interventions cannot yet be considered robust therapeutic adjuncts in patients with GBM (THERApeutic Outcomes Related to Gut microBIOME in Glioblastoma (GBM); Liu et al. 2024; Nourizadeh et al. 2025). Probiotics, prebiotics, diet modification, FMT, and microbial metabolite supplementation may eventually support immune or metabolic modulation, but their safety, timing, donor or strain selection, dosing, durability, and interaction with radiotherapy, temozolomide, corticosteroids, antiepileptic drugs, and immune checkpoint inhibitors remain unresolved (Lei et al. 2025; Liu et al. 2024; Dalile et al. 2019; Baruch et al. 2021).

Microbial metabolites provide one of the most plausible translational routes because they can connect intestinal ecology with systemic immunity, epigenetic regulation, and tumor‐cell biology (Krautkramer et al. 2016; Liu et al. 2023). Sodium butyrate has shown direct antitumor and immune‐modulating effects in preclinical glioma systems, including reduced glioma cell viability, increased apoptosis, altered cell‐cycle regulation, and enhanced anti‐PD‐1 efficacy in orthotopic models (Majchrzak‐Celińska et al. 2021). Polyamine metabolism and microglial fructose metabolism also highlight immune‐metabolic vulnerabilities within the GBM TME (Miska et al. 2021). These pathways are clinically relevant because they suggest that GBM immunosuppression may be shaped by metabolically adapted myeloid and microglial programs, but therapeutic targeting of these mechanisms still requires pharmacokinetic, toxicity, BBB penetration, and patient‐selection studies (Miska et al. 2021; Liu et al. 2023).

Microbial peptide presentation represents another translationally important area. GBM‐specific immunopeptidomic evidence indicates that HLA molecules in GBM tissues and GBM cell lines can present bacteria‐specific peptides, and selected microbial peptides can activate tumor‐infiltrating lymphocytes ex vivo (Naghavian et al. 2023). This finding supports the possibility that microbial peptides may contribute to the GBM antigenic landscape and may inform future vaccine design, microbial‐peptide screening, and personalized immunotherapy (Zhang et al. 2024; Naghavian et al. 2023). The clinical value of this approach will depend on whether reproducible peptide‐HLA complexes can be identified across patient cohorts, whether they generate functional antitumor T‐cell responses, and whether they can be targeted safely without inducing tolerance or off‐target inflammation (Naghavian et al. 2023; Taylor et al. 2021).

Several barriers currently limit clinical translation. Human microbiome composition is shaped by diet, geography, age, antibiotic exposure, corticosteroids, antiepileptic medication, chemotherapy, radiotherapy, comorbidities, hospitalization, and perioperative factors (Lei et al. 2025; Stepanenko et al. 2024). GBM itself introduces additional complexity through tumor heterogeneity, blood‐brain and blood‐tumor barrier disruption, necrosis, corticosteroid‐driven immunosuppression, lymphopenia, and local myeloid dominance (Stepanenko et al. 2024; Stepanenko et al. 2024). These variables make it difficult to distinguish causal microbiome effects from disease‐associated or treatment‐associated microbial changes (Zhang et al. 2022; Lei et al. 2025). Standardization of sample collection, sequencing protocols, contamination control, metabolite quantification, and immune correlates will be necessary before microbiome‐based biomarkers or interventions can be integrated into clinical neuro‐oncology (Eisenhofer et al. 2019; Fierer et al. 2025; THERApeutic Outcomes Related to Gut microBIOME in Glioblastoma (GBM)).

Future studies should move beyond taxonomic description toward causal and spatially resolved mechanisms (Morad et al. 2025; Li et al. 2025). Priority should be given to longitudinal clinical cohorts with matched stool, blood, tumor, and treatment‐response data; patient‐derived glioma models; gnotobiotic and humanized microbiome systems; spatial transcriptomics; immunopeptidomics; metabolomics; and single‐cell immune profiling (THERApeutic Outcomes Related to Gut microBIOME in Glioblastoma (GBM)). Clinical studies should also evaluate how microbiome features interact with corticosteroid exposure, antibiotic timing, temozolomide response, radiation necrosis, immune checkpoint blockade, tumor vaccines, and oncolytic virotherapy (Liu et al. 2024; Stepanenko et al. 2024). The most realistic near‐term clinical goal is not the replacement of standard GBM therapy, but development of microbiome‐informed stratification and rational adjuvant strategies that can be tested in carefully controlled translational studies (THERApeutic Outcomes Related to Gut microBIOME in Glioblastoma (GBM); Lei et al. 2025; Liu et al. 2024) (Table 2).

**TABLE 2:** Translational opportunities, evidence level, and current limitations of microbiome‐related strategies in glioblastoma.

Translational domain	Candidate microbiome‐related feature	Potential clinical relevance	Current level of evidence	Major limitations	Recommended next steps	Ref
Risk association and patient stratification	Gut microbial taxa such as Ruminococcaceae, *Faecalibacterium*, Peptostreptococcaceae, and the *Eubacterium brachy* group	May help generate hypotheses about microbiome‐linked GBM susceptibility and immune‐metabolic phenotypes	Human association and Mendelian randomization evidence	Does not prove causality; affected by geography, diet, host genetics, antibiotic exposure, and statistical model assumptions	Longitudinal metagenomic and metabolomic cohorts with matched clinical variables	(Jiang et al. 2024; Gao et al. 2025)
Non‐invasive biomarkers	Stool microbiome profiles, circulating microbial metabolites, inflammatory signatures	Potential support for prognosis, treatment‐response prediction, or immune phenotyping	Early clinical association and preclinical evidence	No validated GBM‐specific diagnostic microbiome signature; high interindividual variability	Multi‐center validation with standardized sampling and treatment‐response endpoints	(THERApeutic Outcomes Related to Gut microBIOME in Glioblastoma (GBM), Yan et al. 2024)
Tumor‐associated microbial signals	Low‐abundance bacterial DNA, RNA, lipopolysaccharide, spatial bacterial markers	May support tumor immunophenotyping or identification of microbial‐tumor immune interactions	Low‐biomass sequencing, spatial validation, and glioma‐focused multi‐omics evidence	Contamination risk; uncertain microbial viability; limited reproducibility across cohorts	Negative controls, paired tissue analysis, FISH or spatial validation, and functional assays	(Li et al. 2024; Eisenhofer et al. 2019)
Microbial peptide‐HLA presentation	Bacteria‐specific HLA‐bound peptides in GBM tissues and GBM cell lines	May expand antigen discovery for vaccines, T‐cell monitoring, or personalized immunotherapy	GBM‐specific immunopeptidomic and ex vivo TIL activation evidence	Unknown reproducibility, source of peptides, HLA restriction, and safety of amplification	Integrate HLA typing, immunopeptidomics, TCR sequencing, and functional T‐cell assays	(Kalaora et al. 2021; Naghavian et al. 2023)
Metabolite‐based immune modulation	SCFAs, especially butyrate; tryptophan‐derived metabolites; polyamines	May influence epigenetic regulation, glial activity, T‐cell function, and myeloid suppression	Preclinical glioma and broader neuroimmune evidence	Dose, delivery, BBB penetration, pharmacokinetics, and patient selection remain unresolved	Controlled metabolomic studies and preclinical combination testing with standard therapy	(Majchrzak‐Celińska et al. 2021; Li et al. 2025)
Myeloid and microglial metabolic targeting	Polyamine metabolism in TAMCs; GLUT5‐dependent fructose metabolism in microglia	May identify metabolic vulnerabilities that sustain GBM immunosuppression	Strong mechanistic preclinical evidence with human‐sample support	Therapeutic targetability, CNS safety, and clinical biomarkers remain uncertain	Validate druggable targets, pharmacodynamics, and immune correlates in patient‐derived models	(Miska et al. 2021; Billingham et al. 2026)
Microbiome modulation	Diet, prebiotics, probiotics, FMT, microbial consortia	May support immune homeostasis or improve response to immunotherapy as an adjunctive strategy	Mostly preclinical glioma evidence; stronger clinical evidence exists in non‐CNS tumors	No established GBM treatment protocol; safety and timing uncertain	Carefully controlled early‐phase trials with immune, metabolic, and survival endpoints	(Fan et al. 2022; D'Alessandro et al. 2020)
Therapy‐response modification	Microbiome interactions with temozolomide, radiotherapy, corticosteroids, antibiotics, immune checkpoint blockade, vaccines, and oncolytic virotherapy	May explain heterogeneity in treatment response and toxicity	Emerging preclinical and observational evidence	Confounding by medication exposure, tumor burden, diet, and hospitalization	Prospective sampling during treatment and mechanistic analysis of responders versus non‐responders	(THERApeutic Outcomes Related to Gut microBIOME in Glioblastoma (GBM); Patrizz et al. 2020)
Clinical implementation	Integrated microbiome, metabolome, immune, and imaging‐based stratification	May support future personalized neuro‐oncology pipelines	Conceptual and early translational stage	No validated standard workflow; regulatory and manufacturing barriers for live biotherapeutics	Multi‐omic clinical studies, harmonized protocols, and regulatory‐grade intervention design	(Fierer et al. 2025, Xie et al. 2025)

## 9 Conclusion

GBM remains one of the most challenging diseases in neuro‐oncology because of its diffuse invasion, profound immune evasion, metabolic adaptability, and limited responsiveness to current therapeutic strategies. Accumulating evidence indicates that the gut microbiota, microbiota‐associated metabolites, and systemic neuroimmune signaling may influence glioma‐relevant inflammatory pathways, although most mechanistic evidence remains preclinical or early translational. The microbiome may affect GBM biology through several interconnected routes, including modulation of peripheral immune tone, microglial maturation, BBB regulation, T‐cell polarization, and metabolite‐driven epigenetic or immunometabolic signaling. Recent studies also suggest that metabolic programs involving sodium butyrate, polyamine metabolism in TAMCs, and GLUT5‐dependent fructose metabolism in microglia may shape GBM immune suppression and antitumor immune activity.

Evidence for tumor‐associated microbial signatures and microbial peptide‐HLA presentation has introduced an additional layer of complexity to GBM immunobiology. However, these findings should be interpreted cautiously because brain tumor specimens are low‐biomass samples, and microbial detection requires stringent contamination control, spatial validation, and functional confirmation. Preclinical models show that microbiome manipulation can alter glioma‐associated immune dynamics and tumor behavior, but translation to patients requires careful evaluation of safety, timing, patient heterogeneity, medication exposure, and treatment interactions.

The most realistic near‐term value of microbiome research in GBM lies in mechanistic discovery, immune‐metabolic stratification, biomarker development, and rational adjuvant strategies rather than the immediate replacement of established treatment. Future studies should prioritize longitudinal clinical cohorts, standardized microbiome and metabolomics workflows, patient‐derived and humanized glioma models, spatially resolved tumor profiling, immunopeptidomics, and single‐cell immune analysis. By integrating microbiome science with precision neuro‐oncology, future research may clarify whether microbiota‐associated signals are causal contributors, adaptive byproducts, or clinically targetable modifiers of GBM progression and therapeutic resistance.

## Author Contributions

**Mehrdad Nourizadeh**: conceptualization, Writing – original draft, Writing – review and editing and supervision. **Tareq Nayef AlRamadneh**: conceptualization, writing – original draft, writing – review and editing. **Renuka Jyothi S**: conceptualization, writing – original draft, writing – review and editing. **Priya Priyadarshini Nayak**: conceptualization, writing – original draft, writing – review and editing. **Anima Nanda**: conceptualization, writing – original draft, writing – review and editing. **Shaker Al‐Hasnaawei**: conceptualization, writing – original draft, writing – review and editing. **Akanksha Bhatt**: conceptualization, writing – original draft, writing – review and editing. **Ashish Singh Chauhan**: conceptualization, writing – original draft, writing – review and editing. **Siya Singla**: conceptualization, writing – original draft, writing – review and editing.

## Funding

The authors have nothing to report.

## Ethics Statement

The authors have nothing to report.

## Consent

The authors have nothing to report.

## Use of Artificial Intelligence

AI‐assisted tools were used only for language editing and improving clarity. All scientific content, references, and conclusions were checked and approved by the authors, who take full responsibility for the manuscript.

## Conflicts of Interest

The authors declare no conflicts of interest.

## Data Availability

Data sharing is not applicable to this article as no datasets were generated or analyzed during the current study.
